# Identification and functional characterization of CD8^+^ T regulatory cells in type 1 diabetes patients

**DOI:** 10.1371/journal.pone.0210839

**Published:** 2019-01-16

**Authors:** Marsha Pellegrino, Antonino Crinò, Manuela M. Rosado, Alessandra Fierabracci

**Affiliations:** 1 Infectivology and Clinical Trials Research Division, Bambino Gesù Children's Hospital, Rome, Italy; 2 Endocrinology Department, Bambino Gesù Children's Hospital, Rome, Italy; 3 Bambino Gesù Children's Hospital, Research Laboratories, Rome, Italy; Mississippi State University, UNITED STATES

## Abstract

Type 1 diabetes is an autoimmune disease where autoreactive T lymphocytes destroy pancreatic beta cells. We previously reported a defect in CD4^+^ Tregs cell proliferation and reduced CD4^+^ Tregs PD-1 expression in patients. Another ‘memory-like’ regulatory subset, CD8^+^ Tregs, evaluated as CD8^+^CD25^+^FOXP3^+^, has recently raised interest for their effective suppressive activity. Different CD8^+^ T cell populations, their proliferation capacity and expression of PD-1 molecule were evaluated by flow-cytometer analysis in newly diagnosed, long-term Type 1 diabetes patients compared to healthy normal donors. Under basal conditions, CD8^+^ Tregs and CD8^+^ Teffs were seemingly represented among study groups while there was evidence of diminished expression of PD-1 in Teff subsets of long-term patients. After 3 days of PMA/ionomycin stimulation, patients CD8^+^ Tregs showed decreased percentage in respect to control group. CD8^+^ Teffs were instead increased in long-term diabetics *versus* controls. PD-1^+^CD8^+^ Tregs were represented at a much lower percentage in long-term diabetic patients, in respect to controls. Importantly, patients CD8^+^ Tregs and CD8^+^ Teffs presented a significant proliferation defect in respect to the control group. In conclusion, our study indicates that a defect of CD8^+^ Tregs is observed in diabetics. This subset could thus represent a novel target of immunotherapy in patients.

## Introduction

Insulin-dependent diabetes mellitus (Type 1 diabetes, T1D) is due to the autoimmune destruction of insulin producing pancreatic islet beta cells by autoreactive effector T lymphocytes [1, 2]. Within its multifactorial pathogenesis, a close interaction of genetic background and environmental agents plays a major role. Establishment of thymic central tolerance in the perinatal age leads to elimination of autoreactive clones. Nevertheless, autoreactive T cells escape to thymic deletion and survive in the circulating peripheral blood (reviewed (rev) in [3, 2]); this implies that the immune system homeostasis is also contributed by peripheral tolerance. Thus, both altered central and peripheral tolerance mechanisms affect the onset and progression of the clinical disease; in particular this implies an altered function of T regulatory cells (Tregs) that play a fundamental role in controlling host immunity to self- or non-self-proteins and infectious agents [2,4].

To date the role of CD4^+^CD25^+^ Tregs in controlling activation of effector autoreactive T lymphocytes in the pathogenesis of autoimmune disorders still requires to be fully elucidated [2,5]. T1D studies in particular yielded conflicting results regarding their frequency and/or number as well as their suppressive function in T1D patients. Indeed, observed discrepancies across different investigations could be due to diverse biological materials employed such as lymphocytes obtained from the peripheral blood or lymph nodes, even from patients at different stage of disease (onset *versus* long-term) or of different ethnic populations [2].

We recently provided evidence for defects of CD4^+^CD25^+^CD127^low^ Tregs in the peripheral blood of T1D patients [2]. Their percentages were significantly higher in basal conditions while that of T effector cells (Teffs) significantly lower in patients than in controls. Tregs were functional in patients as Tregs ratio to Teffs was higher in patients than in controls. Further Treg subsets were characterized based on the expression of programmed-cell death (PD-1). PD-1 or CD279 and its ligand PDL-1 appear to play a significant effect on immune tolerance and cell death. Regarding PD-1/PDL-1 pathway, its regulation affects the function of several immunotypes including Tregs [2,6]. In our study although percentages of total PD-1^+^, PD-1^low^ and PD-1^high^ expressing Tregs were similar in patients and in controls in basal conditions, lower Tregs proliferation was observed upon anti-CD3/CD28 stimulation in diabetics [2]. Furthermore, percentages of total PD-1^+^, PD-1^low^ and PD-1^high^ expressing Tregs subpopulations were lower in diabetics [2].

Recently, investigations aimed to identify and characterize other Tregs populations beside CD4^+^CD25^+^. In this regard, CD8^+^ T cells are recognized as adaptive immunological suppressive effectors in several conditions, i.e. cancer, transplantation, host defense and even autoimmune diseases (rev in [4]). Authors have described also CD8^+^ Tregs, both in mice and humans [7], that remained neglected for long time due to the lack of available unique markers [8]. Nevertheless, several subsets of CD8^+^ Tregs were depicted based on the expression of CD25, CD56 [9], FoxP3, CXCR3, CD122, CD38, CD8αα, CD45RA, CD45RO, LAG-3 and/or HLA-G as well as the absence of CD28 expression and CD127 (rev in [10, 11]). Several mechanisms are involved in CD8^+^ Tregs suppressive function: release of immunosuppressive factors and inhibitory cytokines such as IL-10 or direct lysis of target cells through cell-cell contact [12]. Furthermore, CD39^+^CD26^-^CD8^+^ Tregs release nicotinamide adenine dinucleotide phosphate (NADPH) oxidase 2 containing extracellular vesicles that act by reducing phosphorylation of the T cell receptor (TCR)-associated kinase ZAP70 through reactive oxygen species (ROS) induction. Indeed, CD8^+^ Tregs were able to regulate general immune responses as well as specific autoimmune T cells keeping under control the normal T cell repertoire in the periphery [13].

Few evidences are reported so far for alterations of CD8^+^ suppressive T cells in human autoimmune diseases i.e. rheumatoid arthritis (RA), systemic lupus erythematosus (SLE), ankylosing spondylitis, systemic sclerosis, multiple sclerosis, myasthenia gravis, primary biliary cirrhosis, experimental autoimmune uveoretinitis, Hashimoto’s thyroiditis and Graves’ disease (rev in [14]). Regarding T1D, suppressive CD8^+^ subsets were indeed able to prevent and even reverse T1D in non-obese diabetic (NOD) mice [15] where, in particular, the subset of CD8^+^PD-1^+^ Tregs, expressing the β subunit of IL-2 receptor CD122 instead of CD25, revealed to be functional in ameliorating disease [16]. In a trial employing a modified anti-CD3 monoclonal antibody (mAb) [hOKT3ɣ1 (Ala-Ala)] in T1D patients, Bisikirska et al (2005) [17] originally depicted in responders an increased number and activation of CD8^+^ T cells upon treatment. In particular, anti-CD3 mAb induced the expansion of CD8^+^CD25^+^FoxP3^+^CTLA4^+^ Tregs. This regulatory population was able to inhibit, through contact-dependent mechanism, CD4^+^ T cell responses to the mAb as well as to antigen [17].

Complex interactions are postulated to occur between different subsets of Tregs in order to maintain a balance on the level of tolerance to self thus avoiding altered immunosuppression [18]. It is of interest to note that, specifically in NOD mice, CD4^+^ Th cells and CD4^+^CD25^+^ Tregs would exert opposite roles in the development of memory-like autoregulatory CD8^+^ T cells [18]. To this extent, CD4^+^ Th cells and CD8^+^ Teffs would recruit functional FoxP3^+^ Tregs that, in turn, halt both effector and memory autoregulatory CD8^+^ T cells, thus affecting autoantigen presentation. Current knowledge from NOD mice suggests that autoregulatory CD8^+^ cells arise spontaneously from non-pathogenic low-avidity clones and halt autoantigen-loaded antigen-presenting cells (APCs) in pancreatic lymph nodes [15].

In the light of the foregoing, the aim of this study was to evaluate percentages of CD8^+^ Tregs in the peripheral blood of T1D patients in different phases of disease at onset and during long-term disease in comparison with healthy subjects. Furthermore, we functionally assess these subsets based on the expression of PD-1 molecule.

## Materials and methods

### Subjects

The patient group consisted of 18 newly diagnosed (ND) and 13 long-standing (long-term, LT) T1D patients. To the ND group of patients belong those subjects recruited at the time of T1D diagnosis, while those subjects with at least 10 years of disease belong to the group of LT patients. Patients were recruited at the Department of Endocrinology at Bambino Gesù Children’s Hospital (OPBG) over the past five years. Patients’ sera were tested for diabetes-related autoantibodies (AAbs) i.e. glutamic acid decarboxylase isoform 65 (GADA), protein tyrosine phosphatase insulinoma-associated antigen 2 (IA2) and insulin (IAA) AAbs by radioimmunoassay (RIA), thyroglobulin (Tg), thyroperoxidase (TPO) and tissue transglutaminase (tTGA) AAbs by chemiluminescence (ADVIA Centaur analyzer: Siemens Healthcare, Germany), parietal cell (PCA), adrenal cortex (ACA) and islet cell AAbs by indirect immunofluorescence (IFL). Mean glycated hemoglobin (HbA1c) value of patients was 101.3 mmol/mol for newly diagnosed and 66.5 mmol/mol for long-term subjects (cut-off value 48 mmol/mol), indicating a poor metabolic control, which required insulin therapy adjustments. The control group of 20 HD, without family history for autoimmune diseases and no circulating AAbs, was recruited from the OPBG Blood Transfusion Division. All controls in respect to patients were matched for sex, age, ethnic and geographical origin. Enrolled individuals either patients or controls were unrelated. All subjects were recruited in the investigation after obtaining written informed consent. The study was approved by the local Institutional Review Board (IRB) of the OPBG, which regulates the use of human samples for experimental studies. The written informed consent for the children was obtained from the next of kin. The participants’ consent was recorded using a paper-based inventory system. The IRB approved the consent procedure (1385_OPBG_2017, 14 June 2017).

### Cell preparation

Peripheral blood mononuclear cells (PBMC) were separated by Ficoll-Hypaque (Histopaque, Sigma-Aldrich Chemical: St Louis, MO, USA) from sodium heparinized venous blood samples (5–10 mL). Samples were cryo-preserved in liquid-nitrogen according to standard procedures [2,19].

### Stimulation of PBMC with phorbol myristate (PMA)-ionomycin

Liquid-nitrogen frozen PBMC from healthy donors and from T1D patients were thawed in complete RPMI medium (Gibco RPMI 1640 Medium, ThermoFisher Scientific, Waltham, MA, USA) supplemented with 10% fetal bovine serum (FBS, Hyclone, South Logan, UT, USA), L-glutamine (2mM) (EuroClone S.p.A., MI, Italy) and 1% penicillin/streptomycin (pen/strep) (EuroClone) according to established protocols [19] and centrifuged at 1200 rpm for 5 minutes at room temperature (RT). Cells were cultured in 48 well plates (Falcon, Corning Incorporated, NY, USA), 1.5×10^6^ cells per well in complete RPMI. Subsequently, cells were stimulated with the addition of 7.5 ng/ml phorbol-12-myristate-13-acetate (PMA) (Calbiochem, Merk, Darmstadt, Germany) and 0.8μg/ml Ionomycin (IONO) (Sigma Aldrich, Merk). The cells were incubated for three to five days at 37°C in a humidified atmosphere containing 5% CO_2_. At the end of the incubation period, cells were harvested from culture plates and washed by centrifugation 1200 rpm for 5 minutes in PBS at RT. Subsequently cells were stained for FACS analysis as described below.

### Proliferation assay

In order to assess cell proliferation, before stimulation, PBMC were previously labeled with 0.1 μg/ml final concentration of 5-chloromethyl fluorescein diacetate (CMFDA) (CellTracker, Invitrogen, Molecular Probes, OR, USA) for 30 minutes at 37°C, washed once by centrifugation at 1200 rpm for 5 minutes at RT and cultured at 7.5x10^5^ cells per well in 96 well flat-bottom plates. Subsequently, the cells were stimulated as described above and incubated for three and five days at 37°C in a humidified atmosphere containing 5% CO_2._ Cell proliferation was assessed at three and five days by flow cytometry following the staining procedure described below.

### Flow cytometry analysis (FACS)

In order to analyze untreated PBMC of HD, ND and LT patients under basal conditions, liquid-nitrogen frozen PBMC were thawed in complete RPMI medium and washed once by centrifugation at 1200 rpm for 5 minutes at RT. A total of 1.5×10^6^ cells per sample were subsequently used for antibodies staining procedure. To identify T cell subsets, the cells were stained for 20 minutes at 4°C with the following antibodies: Brilliant Ultraviolet 737 (BUV737) conjugated mouse anti-human CD3 (Clone UCHT1; 1:40 dilution; BD Biosciences, CA, USA); Brilliant Violet 421 (BV421) conjugated mouse anti-human CD25 (Clone M-A251; 1:40 dilution; BD); allophycocyanin (APC) conjugated mouse anti-human CD8 (Clone RPA-T8; 1:10 dilution; BD) and R-phycoerythrin-Cyanine7 (PE-Cy7) conjugated mouse anti-human CD279 (PD-1) (Clone J105; 1:40 dilution; eBioscience, ThermoFisher Scientific). At the end of the incubation period, cells were washed in phosphate-buffered saline (PBS) (EuroClone) 2% FBS by centrifugation at 1200 rpm for 5 minutes at RT. Subsequently, cells underwent procedure for intracellular FoxP3 staining using PE conjugated mouse anti-human FoxP3 antibody (Clone 259D/C7, BD) according to manufactures’ protocol (Human FoxP3 Buffer Set, BD). Based on available literature CD8^+^CD25^+^FoxP3^+^ cells were identified as CD8^+^ Tregs and CD8^+^CD25^-^FoxP3^-^ cells as CD8^+^Teffs [10, 20]. PD-1^+^ cells within the total gate of CD8^+^ Tregs and Teff were identified as CD8^+^ Treg PD-1^+^ cells and CD8^+^ Teff PD-1^+^ cells.

The same procedure was used for the analysis of CD8^+^ Tregs and CD8^+^ Teffs after three days of PMA/ionomycin stimulation. Cells were stained according to the established protocol described above.

To assess cell proliferation of CMFDA labeled CD8^+^ Tregs and CD8^+^ Teffs, an alternative antibody staining was used (*vide infra*), according to literature [19], after three and five days of PMA/ionomycin stimulation. PBMC were harvested from culture plates and washed by centrifugation at 1200 rpm for 5 minutes at RT in PBS. Subsequently, cells were stained for 20 minutes at 4°C with the following antibodies: BUV737 mouse anti-human CD3 (Clone UCHT1; 1:40 dilution; BD), BV421 mouse anti-human CD25 (Clone M-A251; 1:40 dilution; BD), APC mouse anti-human CD8 (Clone RPA-T8; 1:10 dilution; BD), PE-Cyanine7 mouse anti-human CD279 (PD-1) (Clone J105; 1:40 dilution; eBioscience, ThermoFisher Scientific), PE mouse anti-human CD127 (Clone HIL-7R-M21; 1:40 dilution; BD). Afterwards, cells were washed once by centrifugation at 1200 rpm 5 minutes at RT in PBS 2% FBS. Following this staining procedure, CD8^+^CD25^+^CD127^low^ were depicted as CD8^+^ Tregs and CD8^+^CD25^-^CD127^high^ were indicated as CD8^+^ Teffs.

Data were acquired by flow-cytometer Fortessa X-20 analyzer (Becton and Dickinson (BD), Sunnyvale, CA, USA) and analyzed by FACSDiva software (BD Biosciences: San Jose, CA, USA). Dead cells were excluded from the analysis by side/forward scatter gating (S1 Fig). Fifty thousand lymphocytes per sample were analyzed.

### Correlation of main subsets analyzed and HbA1c

In evaluating the functional significance of divergences observed relative to the immune cell subsets under study in HD and T1D patients, a correlation analysis was performed to investigate the relation between the percentages of these subsets and relative HbA1c (mmol/mol) levels, which are indicative of the metabolic control, in both ND and LT T1D patients.

### Statistical analysis

Due to unfeasibility of concurrent intracellular FoxP3 and CMFDA staining, out of the total group of T1D patients 9 ND and 10 LT were dedicated to the intracellular FoxP3 study while 9 ND and 9 LT were used for the CMFDA proliferation assay.

Differences among the various cell populations analyzed between healthy donors, newly diagnosed and long-term patients were tested for statistical significance with the One-way analysis of variance Kruskal-Wallis test and Dunn’s Multiple Comparison post-test. The results were analyzed using GraphPad Prism software version number 5.00 (GraphPad Software: San Diego, CA, USA). A result with *p* < 0.05 was considered statistically significant. The correlation coefficients between percentages of subsets analyzed and metabolic parameter HbA1c was evaluated with Spearman test.

## Results

### Study population

Within the group of ND and LT T1D patients of the present investigation, the mean actual age of ND T1D patients was 10.8 years (ranging from 5 to 14 years; 11 males, 7 females). The mean age at disease onset was 7.7 years (ranging from 6 to 10 years). The mean actual age of LT T1D patients was 23.6 years (ranging from 20 to 30 years; 6 males, 7 females) and the mean duration of the disease was 13.8 years (ranging from 11 to 19 years). The mean age of the HD controls was 23 years (ranging from 18 to 30 years). Demographic and clinical characteristics of patients are shown in Table 1 and Table 2. In addition to T1D (Table 1), 1 newly diagnosed patient has developed autoimmune thyroid disease (AT). The same patient presented associated celiac disease (CD) and no other pathologies were found in the ND group of patients. Further, in addition to T1D (Table 2), 5 long-term patients have developed also AT (autoimmune polyglandular syndrome Type 3 variant, APS3v); of these 3 were affected by Hashimoto’s thyroiditis (HT), confirmed by the presence of circulating Tg and TPO AAbs and echography pattern of diffuse hypoechogenicity, one patient had developed HT and vitiligo and one Basedow’s disease.

**Table 1:** Demographic, clinical, laboratory and metabolic characteristics of the ND T1D patients recruited for the study.

Pt	Sex	Age of disease onset	Associated diseases	Islet- Related AAbs	Other AAbs	HbA1c
1	F	8		GADA:**5**; IAA:4; IA2:**24**	TPO<28.0; Tg<20.0 UI/mL; tTGA:2.7 CU.	**82**
2	M	8		GADA:0.4; IAA:**15**; IA2:**43**	TPO<28.0; Tg<20.0 UI/mL; tTGA:**27.3** CU.	**82**
3	M	7	AT; CD	GADA:**4**; IAA:2.4; IA2:1.1	TPO**>1300**; Tg:**57.5**UI/mL; tTGA:5 CU.	**86**
4	M	8		GADA:0.2; IAA:1; IA2:0.1	TPO:**73.02**; Tg<20.0 UI/mL; tTGA:13.1 CU.	**100**
5	F	9		GADA:**2.2**; IAA:5; IA2:0.1	TPO<28.0; Tg<20.0 UI/mL; tTGA:0.7 CU.	**96**
6	F	4		GADA:0.7; IAA:6; IA2:**9.2**;	TPO<28.0; Tg<20.0 UI/mL; tTGA:2.4 CU.	**104**
7	M	6		GADA:**3.9**; IAA:6.3; IA2:**57**	TPO:43.2; Tg<20.0 UI/mL; tTGA:0.8 CU.	**100**
8	F	9		GADA:0.1; IAA:6.3; IA2:**4.8**	TPO:33.3; Tg<20.0 UI/mL; tTGA:**30.1** CU.	**143**
9	M	10		GADA:**12**; IAA:5; IA2:**4**	TPO:32; Tg<20.0 UI/mL; tTGA:**47.9** U/mL.	**142**
10	F	8		GADA:**1.2**; IAA:**8**; IA2:0.4	TPO<28.0; Tg<20.0 UI/mL; tTGA:0.2 U/mL.	**115**
11	F	9		GADA:**17**; IAA:6; IA2:**18**	TPO:36.9; Tg<20.0 UI/mL; tTGA:0.2 U/mL.	**117**
12	M	7		IAA:**12**; IA2:31	TPO:47.5; tTGA:0.2 U/mL	**105**
13	M	6		GADA:0.5; IAA:**8**; IA2:**8.1**	TPO:41.9; Tg<20.0 UI/mL; tTGA:0.2 U/mL.	**77**
14	M	8		GADA:**11**; IAA:**7**; IA2:**12**	TPO<28.0; Tg<20.0 UI/mL; tTGA<1.9 CU.	**111**
15	F	9		GADA:**48**; IAA:**8**; IA2:**7.7**	TPO:41; Tg<20.0 UI/mL; tTGA<1.9 CU.	**115**
16	M	7		GADA:0.1; IAA:**7**; IA2:0.1	TPO<28.0; Tg<20.0 UI/mL; tTGA<1.9 CU.	**84**
17	M	7		GADA:**30.7**; IAA:**15**; IA2:**24.3**	TPO:36.3; Tg<20.0 UI/mL; tTGA:**431.4** CU.	**114**
18	M	9		GADA:0.7; IAA:**8.7**;IA2:0.5	TPO:**71.8**; Tg<20.0 UI/mL; tTGA:0.2 U/Ml.	**51**

Islet- related AAbs reference values: glutamic acid decarboxylase isoform 65 (GADA) <0.90 Units/milliliter (U/mL); protein tyrosine phosphatase insulinoma-associated antigen 2 (IA2) < 1.1 U/mL and insulin (IAA) <6.40%; other AAbs reference values: TPO <60 U/mL; tTGA: <20 Chemiluminescent Units (CU) or <4 U/mL. HbA1c reference value <48mmol/mol. Pathological values are indicated in bold. Pt = patient.

**Table 2:** Demographic, clinical, laboratory and metabolic characteristics of the LT T1D patients recruited for the study.

Pt	Sex	Age of Disease Onset	Actual Age	Duration of Disease at referral	Associated Diseases	Islet- Related AAbs	Other AAbs	HbA1c
19	M	8	23	13		GADA:0.7; IA2:**7.5**	TPO< 28.0; Tg<20.0 UI/mL; tTGA:0.7 CU.	**62**
20	F	6	23	15		GADA:0.2; IAA:**44**; IA2:0.1	TPO< 28.0; Tg<20.0 UI/mL; tTGA:1 CU.	**57**
21	F	9	24	13	AT	GADA:0.1; IA2:0.4	TPO**>1300**; Tg<20.0 UI/mL; tTGA:16.2 CU.	**53**
22	F	14	30	13		GADA:0.6; IAA:5.2; IA2:0.7	TPO:37.3; Tg<20.0 UI/mL; tTGA:0.4 CU.	**64**
23	M	7	23	14	AT	GADA:0.1; IAA:**10**; IA2:0.1	TPO:**124.3**; Tg:**98** UI/mL; tTGA:1.85 CU.	**84**
24	M	5	22	14		GADA:0,3; IA2:**4**	TPO<28.0; Tg<20.0 UI/mL; tTGA:0.8 CU.	**73**
25	M	3	24	19	Basedow	GADA:0.1; IAA:**14**; IA2:0.6	TPO<28.0; Tg<20.0 UI/mL; tTGA:16.3 CU.	**72**
26	F	5	21	13		GADA:0.3; IAA:**10**; IA2:0.1	TPO<28.0; Tg<20.0 UI/mL; tTGA:7.6 CU.	**61**
27	F	4	21	12	AT; Vitiligo	GADA:0.1; IA2:**2.8**	TPO:**33.4**; Tg<20.0 UI/mL; tTGA:0.2 U/mL; ACA:pos.	**90**
28	F	4	22	13	AT	GADA:0.6; IAA:**93**; IA2:**3.8**	TPO**>1300**; Tg:**147** UI/mL; tTGA:0.2 U/mL.	**64**
29	M	12	28	11		GADA:**2.9**; IA2:0.1	TPO:31.1; Tg<20.0 UI/mL; tTGA:0.3 U/mL.	**49**
30	F	4	20	13	AT	GADA:0.1; IAA:**8**; IA2:**5.5**	TPO:**274.8**; Tg<20.0 UI/mL; tTGA:**26.8** CU.	**73**
31	M	7	26	17		GADA:0.2; IAA:**35**; IA2:0.4	TPO<28.0; Tg<20.0 UI/mL; tTGA< 1.9 CU.	**63**

### Analysis of CD8^+^ Treg and CD8^+^ Teff cell subsets and their PD-1 expression under basal conditions

#### Similar frequencies of CD8^+^ Treg and CD8^+^ Teff subsets in PBMC of T1D patients and controls

**Fig 1:**
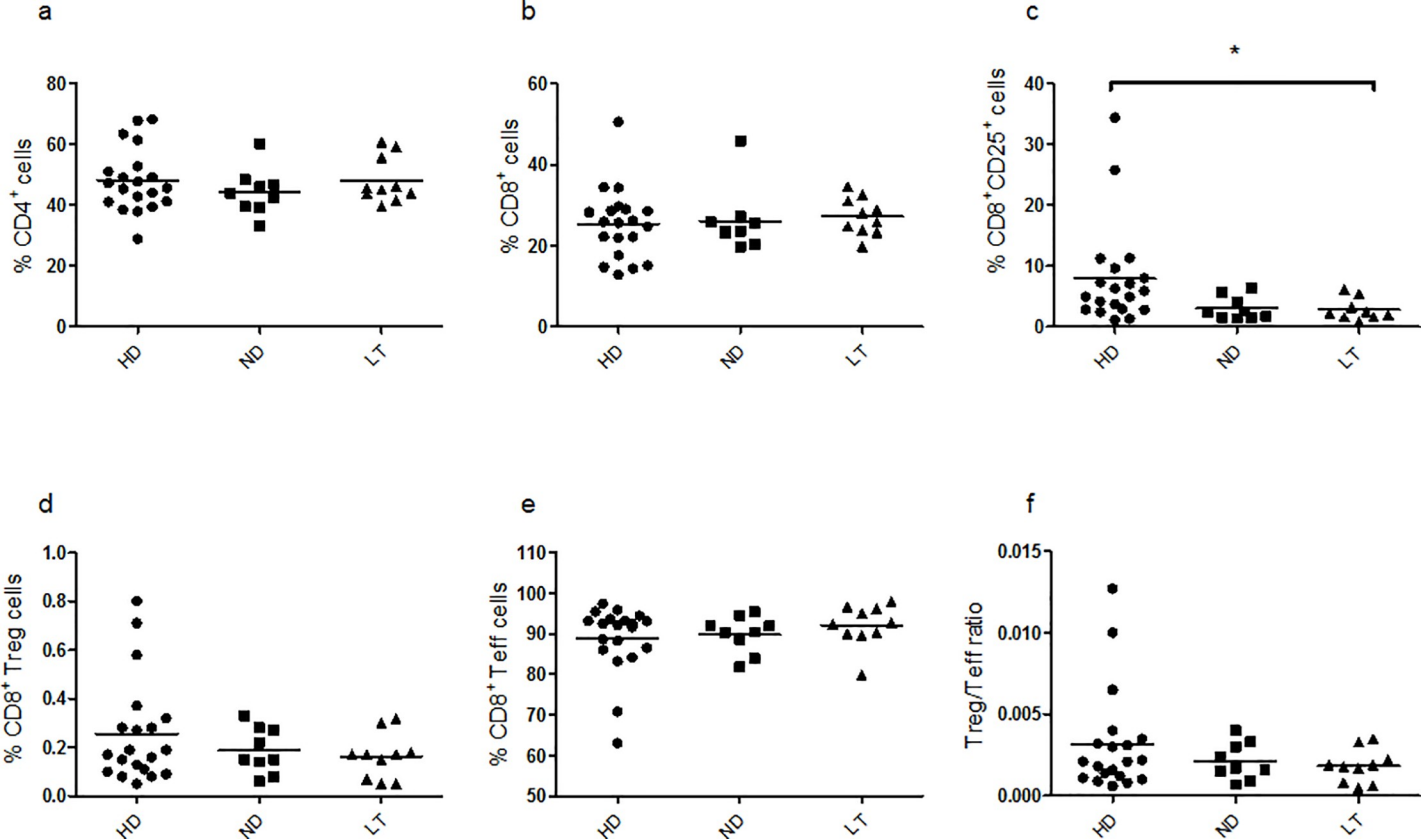
Frequency of cell populations relative to HD, ND and LT T1D patients under basal conditions. Frozen stocked PBMC samples were analyzed by flow-cytometry analysis to estimate respective frequency of CD4^+^ (**a**), CD8^+^ (**b**), CD8^+^CD25^+^ (**c**), CD8^+^CD25^+^Foxp3^+^ Treg (**d**), CD8^+^CD25^-^Foxp3^-^ Teff (**e**) cells and the Treg/Teff ratio (**f**). In all graphs, horizontal lines represent the mean frequency and each symbol shows an individual. Black circles depict HD, square dots represent ND T1D patients and black triangles associate to LT T1D patients. Significance was calculated in relation to the control group using as statistical test the One-way analysis of variance Kruskal-Wallis test and Dunn’s Multiple Comparison post-test. In this and following figures * p<0.05, **p<0.01, and ***p<0.001. For the investigation present in figure, 20 HD, 9 ND and 10 LT samples were studied.

Under basal conditions, percentages of CD4^+^ and CD8^+^ subsets showed no difference in the ND and LT T1D patients *versus* control subjects (Fig 1A, Kruskal–Wallis one-way analysis of variance p = 0.5954; 1b, Kruskal–Wallis one-way analysis of variance p = 0.4756). The percentage of CD8^+^CD25^+^ cells was significantly lower in LT T1D patients than in controls (Fig 1C, Kruskal–Wallis one-way analysis of variance p = 0.0144). The populations corresponding to CD8^+^ Tregs (CD8^+^CD25^+^FoxP3^+^) and CD8^+^ Teffs (CD8^+^CD25^-^FoxP3^-^) were similarly represented among the groups under study, displaying no significant difference (Fig 1D, Kruskal–Wallis one-way analysis of variance p = 0.6271; 1e, Kruskal–Wallis one-way analysis of variance p = 0.4612 and 1f, Kruskal–Wallis one-way analysis of variance p = 0.6539).

#### PD-1 frequency is lower in CD8^+^ Teff of LT T1D patients *versus* controls

**Fig 2:**
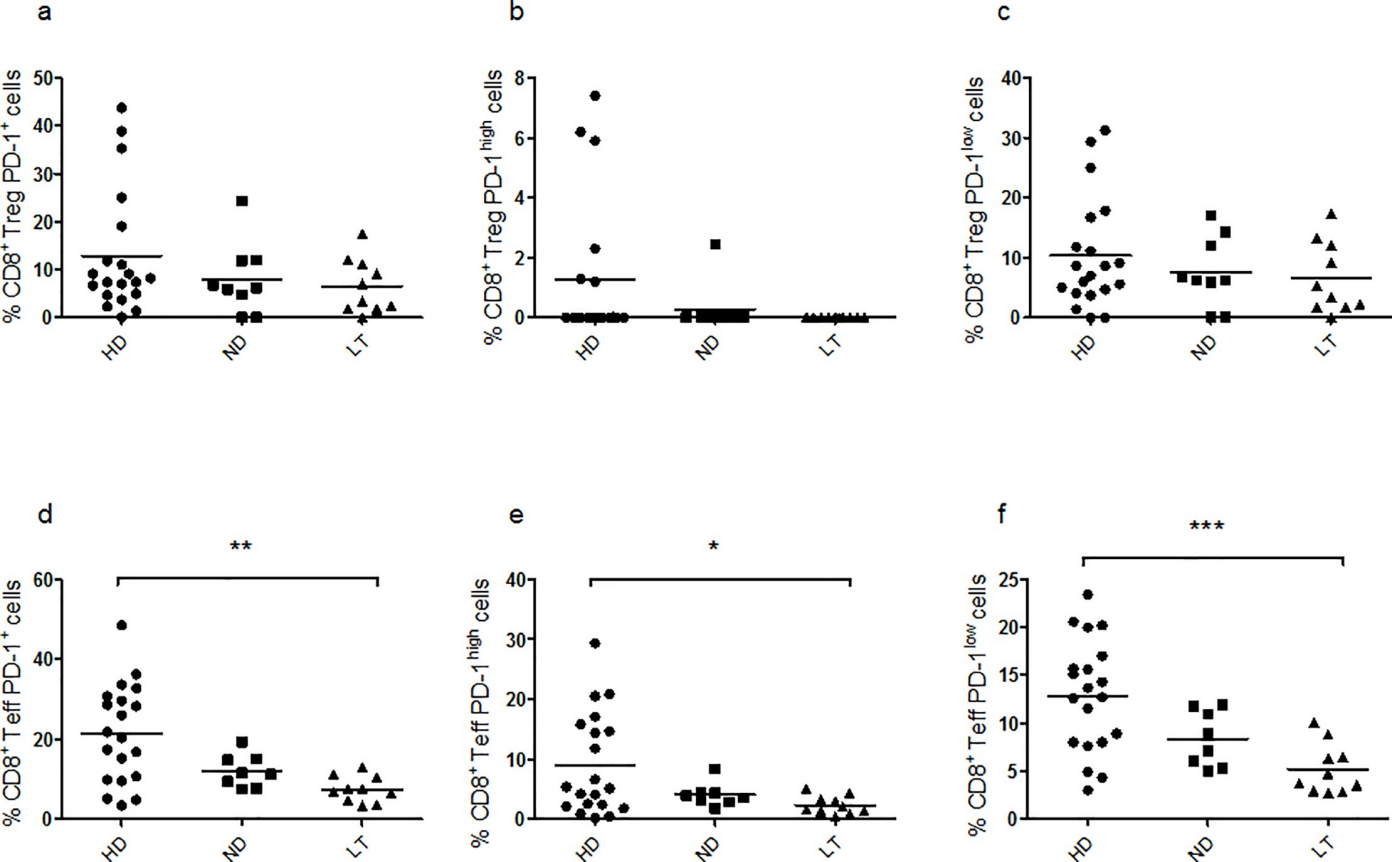
Frequency of CD8^+^ PD-1^+^ subsets under basal conditions. Graphs show the percentages of CD8^+^ Tregs expressing PD-1 (**a**), PD-1^high^ (**b**), PD-1^low^ (**c**), and the percentages of CD8^+^ Teffs expressing PD-1 (**d**), PD-1^high^ (**e**) and PD-1^low^ (**f**). For the investigation present in figure, 20 HD, 9 ND and 10 LT samples were studied.

Under basal condition, no difference in the frequency of total PD-1 (Fig 2A, Kruskal–Wallis one-way analysis of variance p = 0.4233), PD-1^high^ (Fig 2B, Kruskal–Wallis one-way analysis of variance p = 0.1013) and PD-1^low^ (Fig 2C, Kruskal–Wallis one-way analysis of variance p = 0.6449) was observed in CD8^+^ Tregs, while, in CD8^+^ Teffs, the same PD-1 frequency, either total (Fig 2D, Kruskal–Wallis one-way analysis of variance p = 0.0020), PD-1^high^ (Fig 2E, Kruskal–Wallis one-way analysis of variance p = 0.0445) and PD-1^low^ (Fig 2F, Kruskal–Wallis one-way analysis of variance p = 0.0009) appeared significantly lower in the LT T1D PBMC in respect to control PBMC.

### Analysis of CD8^+^ Treg and CD8^+^ Teff cell subsets and their PD-1 expression upon PMA-ionomycin stimulation

#### Lower frequencies of CD8^+^ Tregs and higher frequencies of CD8^+^ Teffs in LT T1D patients

**Fig 3:**
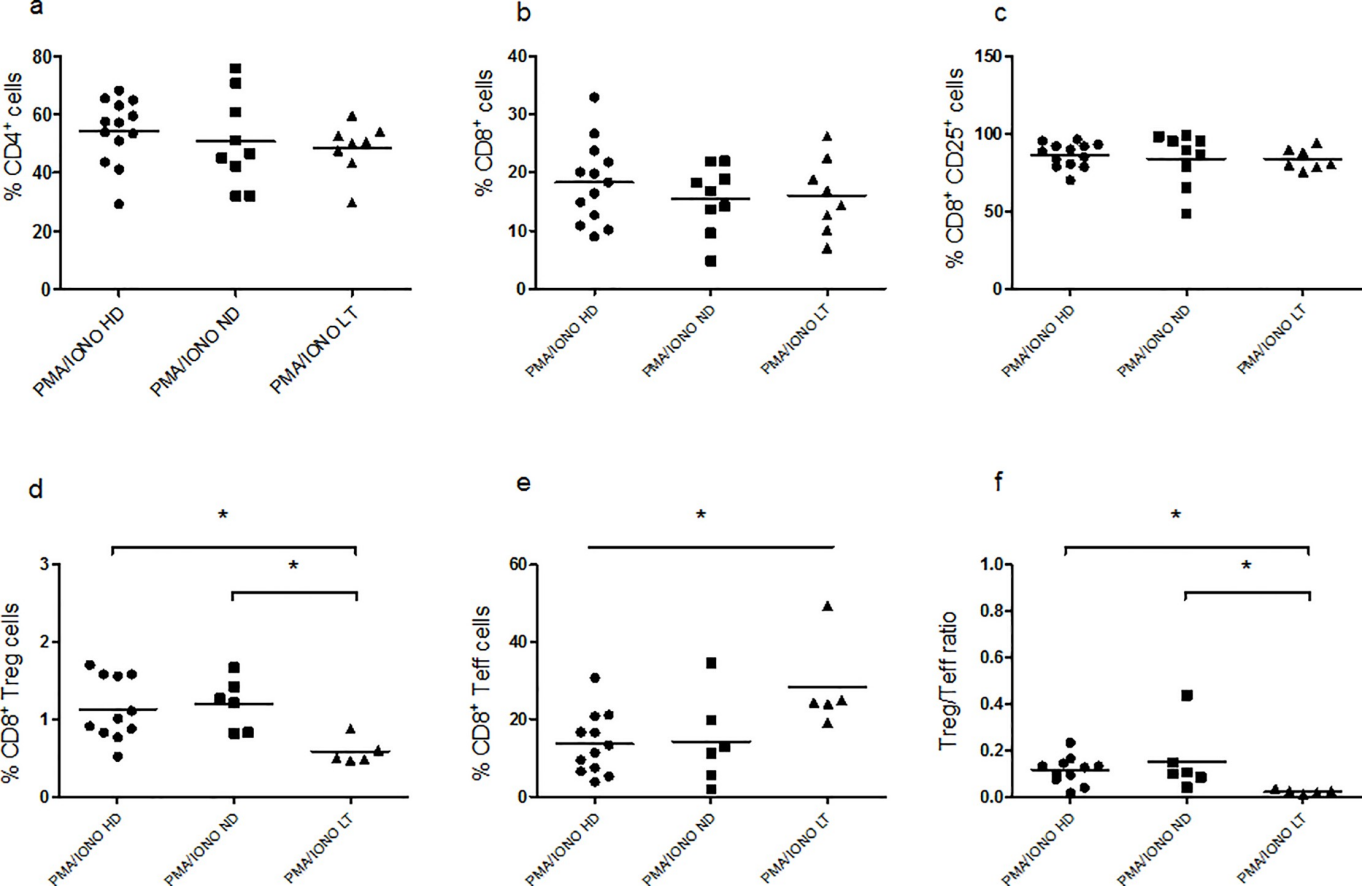
Frequency of cell populations relative to HD, ND and LT T1D patients upon three days of PMA/ionomycin stimulation. Flow-cytometry analysis of healthy donor and patients PBMC following three days of PMA/ionomycin stimuli. Graphs show the percentage of CD4^+^ (**a**), CD8^+^ (**b**), CD8^+^CD25^+^ (**c**), CD8^+^CD25^+^Foxp3^+^ Treg (**d**), CD8^+^CD25^-^Foxp3^-^ Teff (**e**) cells and the Treg/Teff cell ratio (**f**). For the investigation present in figure, 13 HD, 9 ND and 8 LT samples were studied.

After three days of PMA-ionomycin stimulation, no significant difference can be inferred among the three study groups regarding the CD4^+^ (Fig 3A, Kruskal–Wallis one-way analysis of variance p = 0.4139), CD8^+^ (Fig 3B, Kruskal–Wallis one-way analysis of variance p = 0.6981) and CD8^+^CD25^+^ cell populations (Fig 3C, Kruskal–Wallis one-way analysis of variance p = 0.5822). However, we observed that the CD8^+^ Treg population was represented at a much lower percentage in LT T1D PBMC in respect to control subjects and ND T1D PBMC (Fig 3D, Kruskal–Wallis one-way analysis of variance p = 0.0182). The CD8^+^ Teffs, on the other hand, showed increased percentages in LT T1D patients in respect to controls (Fig 3E, Kruskal–Wallis one-way analysis of variance p = 0.0476). As a consequence, the CD8^+^ Treg/Teff cell ratio was much lower in this group of LT patients in respect to controls and also to the ND patients (Fig 3F, Kruskal–Wallis one-way analysis of variance p = 0.0102). Of note, the same cells, kept for three days in the unstimulated (RPMI) culture condition (S2A–S2D Fig) did not show any difference in the percentage relative to the subsets analyzed among the three study groups: CD4^+^ (S2A Fig, Kruskal–Wallis one-way analysis of variance p = 0.2789), CD8^+^ (S2B Fig, Kruskal–Wallis one-way analysis of variance p = 0.4570) CD8^+^ Treg (S2C Fig, Kruskal–Wallis one-way analysis of variance p = 0.7799) and CD8^+^ Teff cells (S2D Fig, Kruskal–Wallis one-way analysis of variance p = 0.0962).

#### Total PD-1 and PD-1^high^ frequency is decreased in CD8^+^ Tregs of LT T1D patients than in controls

**Fig 4:**
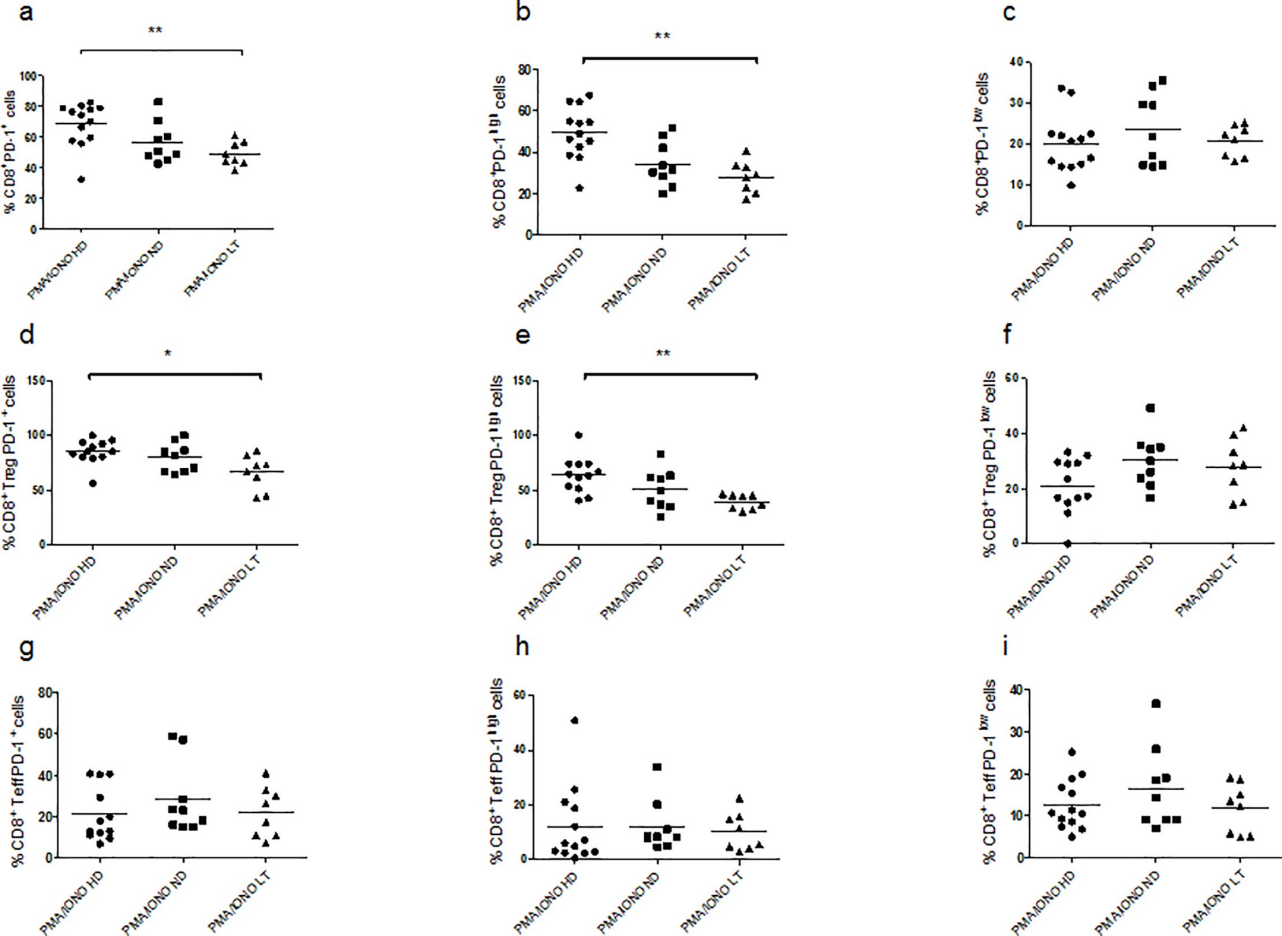
Frequency of CD8^+^ PD-1^+^ subsets upon three days of PMA/ionomycin stimulation. Graphs show the percentages of CD8^+^ cells expressing PD-1 (**a**), PD-1^high^ (**b**), PD-1^low^ (**c**), the percentages of CD8^+^ Tregs expressing PD-1 (**d**), PD-1^high^ (**e**) and PD-1^low^ (**f**) and the percentages of CD8^+^ Teffs expressing PD-1 (**g**), PD-1^high^ (**h**) and PD-1^low^ (**i**). For the investigation present in figure, 13 HD, 9 ND and 8 LT samples were studied.

The analysis of PD-1 expression after three days of PMA-ionomycin stimulation, revealed a significant lower percentage of CD8^+^PD-1^+^ cells and in particular CD8+PD-1^high^ cells in the LT T1D group analyzed in respect to the control group (Fig 4A, Kruskal–Wallis one-way analysis of variance p = 0.0099; 4b, Kruskal–Wallis one-way analysis of variance p = 0.0018), while there was no difference in the CD8^+^PD-1^low^ percentages among the three study groups (Fig 4C, Kruskal–Wallis one-way analysis of variance p = 0.5888).

Regarding CD8^+^ Tregs, the percentages of PD-1^+^ cells was lower in the LT T1D group of patients in respect to the control subjects, in particular, the percentages relative to total PD-1 and PD-1^high^ (Fig 4D, Kruskal–Wallis one-way analysis of variance p = 0.0397; 4e, Kruskal–Wallis one-way analysis of variance p = 0.0054; 4f, Kruskal–Wallis one-way analysis of variance p = 0.1608).

#### PD-1 frequency in similar in CD8^+^ Teffs of T1D patients and controls

No significant difference was revealed in the frequency of PD-1 in the CD8^+^ Teffs among the three study groups after three days of PMA-ionomycin stimulation (Fig 4G, Kruskal–Wallis one-way analysis of variance p = 0.4816; 4h, Kruskal–Wallis one-way analysis of variance p = 0.6602; 4i, Kruskal–Wallis one-way analysis of variance p = 0.5916).

### Analysis of proliferation of CD8^+^ Treg and CD8^+^ Teff cell subsets

#### Proliferation of CD8^+^ Tregs is impaired significantly in ND T1D patients after 3 day-stimulus

**Fig 5:**
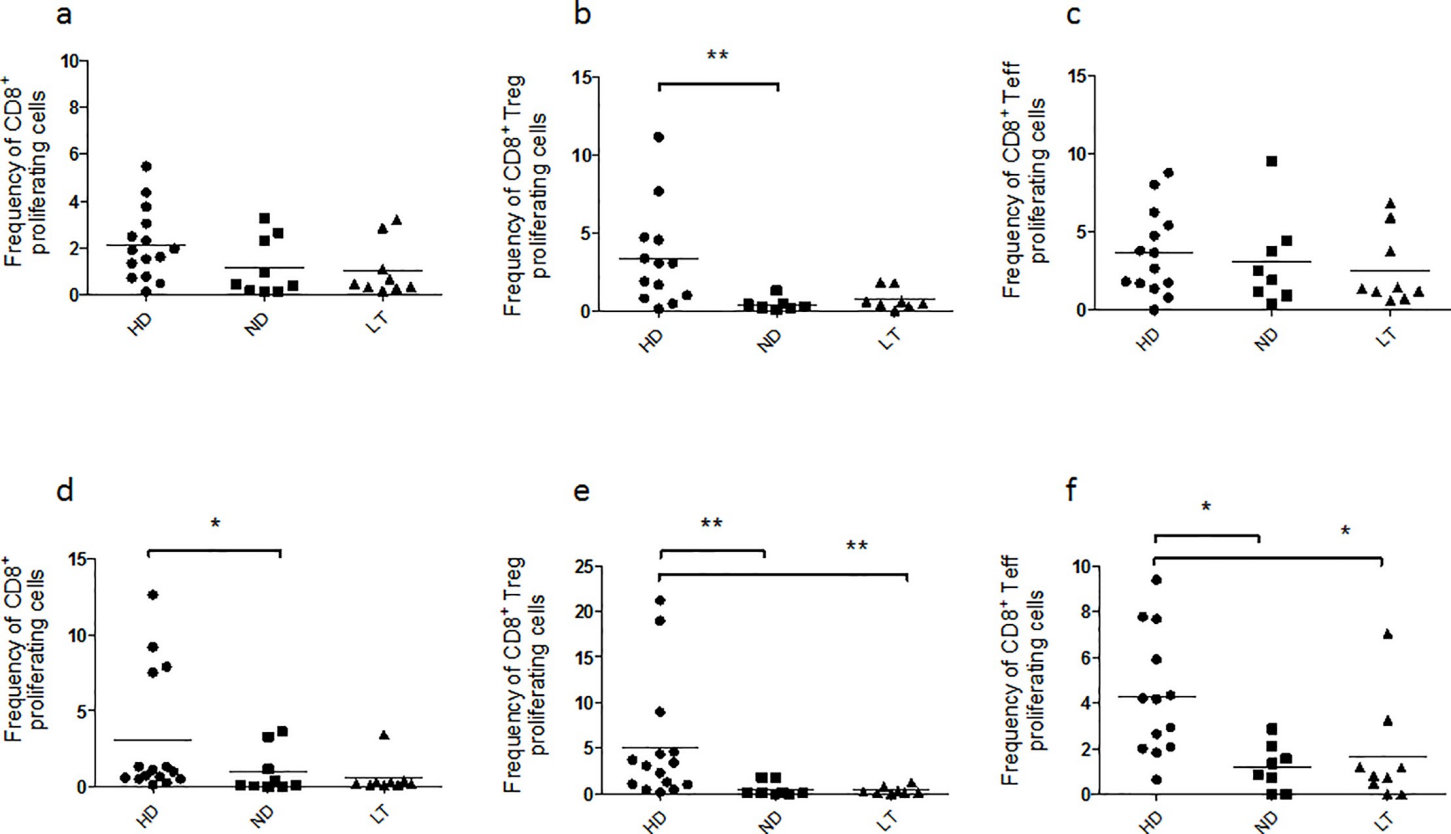
Proliferative responses of cell subsets under study in HD, ND and LT T1D patients after three days of PMA/ionomycin stimulation. CMFDA-labeled PBMC from healthy controls and T1D patients were stimulated with PMA/ ionomycin for three and five days and subsequently stained for flow-cytometry analysis. Graphs show the frequency of CD8^+^, CD8^+^ Treg, CD8^+^ Teff proliferating cells after 3 (**a-c**) and 5 days (**d-f**) of PMA/ionomycin stimulation. Proliferation was evaluated as percentage of CMFDA-low cells relative to the subset analyzed after stimulation over the percentage of CMFDA-low cells of the same subset in RPMI unstimulated cultures. For the investigation present in figure, 14 HD, 9 ND and 9 LT samples were studied.

PMA-ionomycin administration induced T cell proliferation in healthy controls and T1D patients both after three (S3A Fig, Kruskal–Wallis one-way analysis of variance p = 0.1713; b, Kruskal–Wallis one-way analysis of variance p = 0.0704) and five days of stimulation (S3C Fig, Kruskal–Wallis one-way analysis of variance p = 0.0623; d, Kruskal–Wallis one-way analysis of variance p = 0.0553). Upon three days of stimuli (Fig 5A–5C), the proliferation of CD8^+^ cells (Fig 5A, Kruskal–Wallis one-way analysis of variance p = 0.0914) did not show significant differences between the groups of patients analyzed and the controls. However, the frequency of proliferating CD8^+^ Tregs (Fig 5B) appeared lower in both groups of T1D patients in respect to controls but it revealed to be significantly diminished especially in the ND T1D PBMC (Fig 5B, Kruskal–Wallis one-way analysis of variance p = 0.0029). On the other hand, the proliferation of CD8^+^ Teffs after three days showed no significant difference among the three groups under analysis (Fig 5C, Kruskal–Wallis one-way analysis of variance p = 0.4742).

#### Proliferation of CD8^+^ Tregs and CD8^+^ Teffs is impaired in T1D patients after 5 day-stimulus

Prolonged stimulation with PMA-ionomycin for five days (Fig 5D–5F) showed that the frequency of proliferating CD8^+^ cells was decreased in ND T1D PBMC in respect to controls (Fig 5D, Kruskal–Wallis one-way analysis of variance p = 0.0152). Moreover, the proliferative response of CD8^+^ Tregs of both groups of T1D patients is notably lower in respect to the control group (Fig 5E, Kruskal–Wallis one-way analysis of variance p = 0.0007). This result was further observed for the CD8^+^ Teff population (Fig 5F, Kruskal–Wallis one-way analysis of variance p = 0.0051).

### Correlation between main analysed subsets and metabolic marker of disease HbA1c in patients

#### Analysis under basal conditions

No significant correlation was observed between percentages of CD8^+^ Treg cells (relative to Fig 1D) and values of HbA1c in both ND (S4A Fig) and LT T1D patients (S4B Fig). No statistical significance was also observed analyzing percentages of CD8^+^ Treg PD-1^+^ cells in ND and LT T1D patients (S5A and S5B Fig) and percentages of CD8^+^ Teff PD-1^+^ cells in ND and LT T1D patients (S5C and S5D Fig).

#### Analysis after PMA/ionomycin stimulation

**Fig 6:**
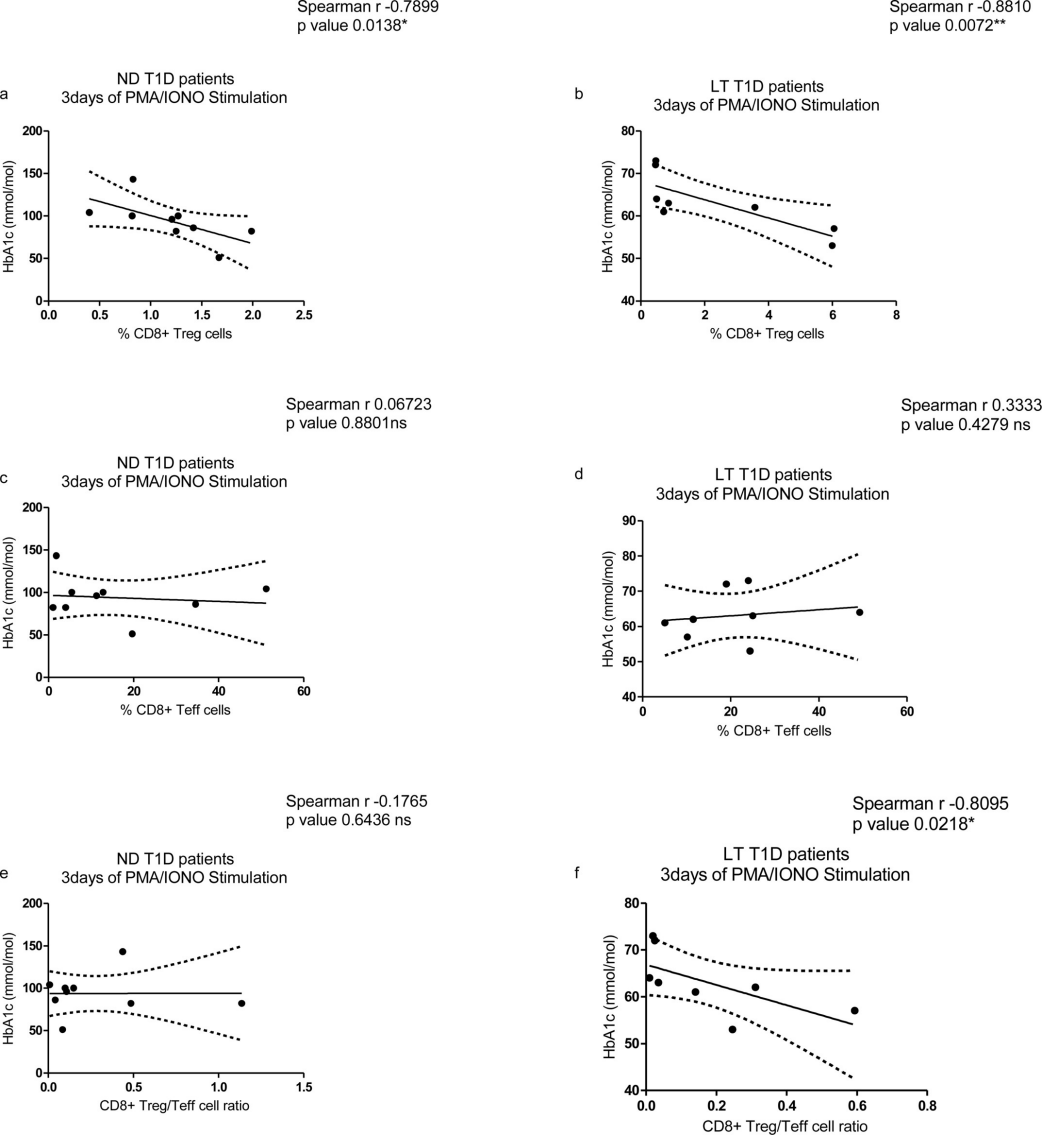
Correlation study between CD8^+^ Treg and Teff cell subsets after PMA/ionomycin stimulation and HbA1c levels. (**a**) Analysis performed for percentages of CD8^+^ Treg cells in ND T1D and (**b**) LT T1D patients; (**c**) Analysis performed for percentages of CD8^+^ Teff cells in ND T1D and (**d**) LT T1D patients. (**e**) Analysis performed for CD8^+^ Treg/Teff cell ratio in ND T1D and (**f**) LT T1D patients. For the investigation present in figure, 9 ND and 9 LT samples were studied.

After 3 days of PMA/ionomycin stimulation a significant inverse correlation was observed between percentages of CD8^+^ Treg cells and HbA1c levels in both ND (Fig 6A) and LT T1D patients (Fig 6B). This suggests that low percentages of CD8^+^ Treg cells are indicative of a worse metabolic control. No significant correlation was found between HbA1c values and percentages of CD8^+^ Teff cells after 3 days of PMA/ionomycin stimulation in ND (Fig 6C) and LT T1D patients (Fig 6D). Regarding CD8^+^ Treg/Teff cell ratio after stimulation, a significant correlation was not observed for ND (Fig 6E) while it was evident for LT T1D patients (Fig 6F).

**Fig 7:**
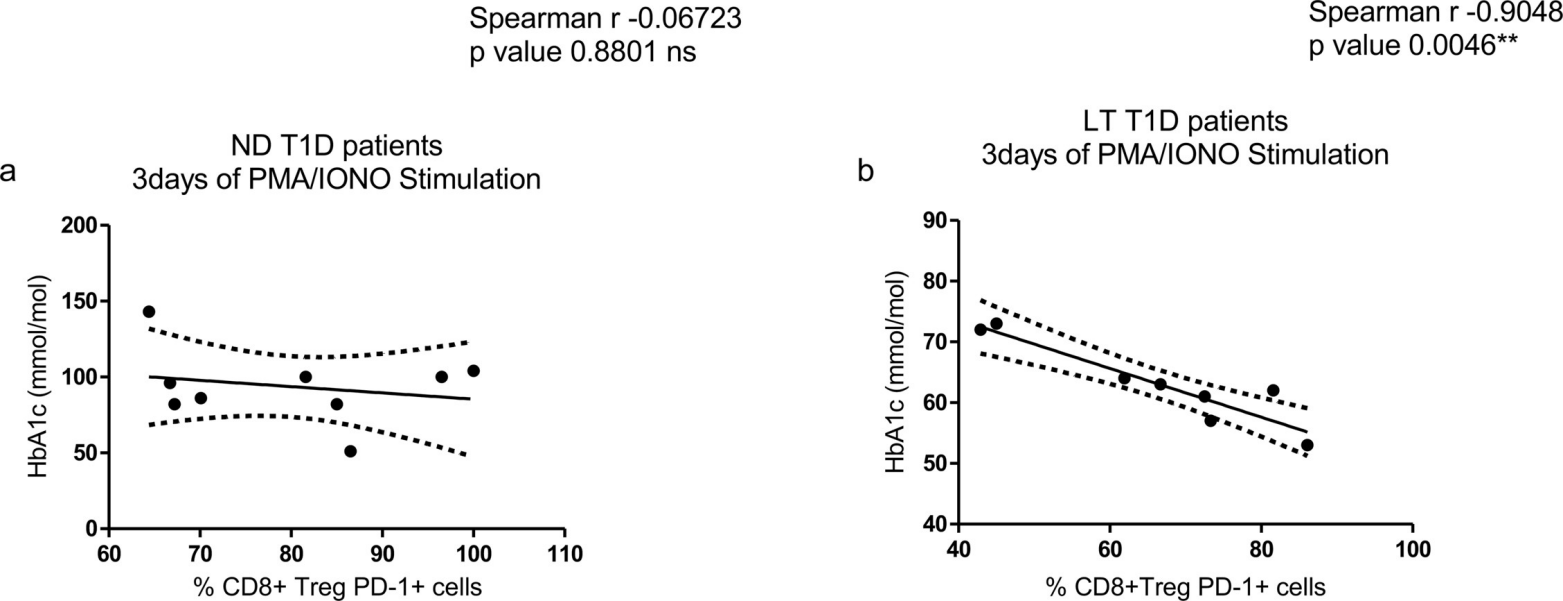
Correlation study between percentages of CD8^+^ Treg PD-1^+^ cells after PMA/ionomycin stimulation and HbA1c levels. (**a**) Analysis performed in ND T1D and (**b**) LT T1D patients. For the investigation present in figure, 9 ND and 9 LT samples were studied.

Moreover, the study did not reveal a significant correlation between percentages of CD8^+^ Treg PD-1^+^ cells after stimulation and HbA1c levels in ND T1D patients (Fig 7A) while it showed a significant inverse correlation in LT T1D patients (Fig 7B). Overall these data further suggest that low percentages of CD8^+^ Tregs and CD8^+^ Treg PD-1^+^ cells correlate with a worse metabolic control of disease in LT patients.

## Discussion

Tregs are known to counteract autoreactive T cells and induce immune tolerance, through dampening inflammation. Regulatory mechanisms beyond their effects on T, B, natural killer (NK) and NKT cells are cell-to-cell-contact, secretion of immunosuppressive cytokines, effects on APCs, and competition for growth factors.

In the light of the foregoing, novel immunotherapies could be exploited to target specifically Tregs in the perspective to avoid autoimmune disease onset and progression. For long time the only proposed treatment option was halting Teffs function, however, nowadays activating/expanding Tregs is a novel avenue that may lead to improved outcomes with enhanced safety [21] (rev in [22]).

In recent years, the complexity of natural and adaptive Tregs has therefore been the object of several studies [4,23] (*vide supra*). Natural CD4^+^ Tregs arise in the thymus, express both CD25 and the master regulator of their differentiation and function Foxp3. CD4^+^CD25^+^FoxP3^+^ Tregs are indeed the subtype more extensively investigated. Adaptive Tregs develop in the periphery following self or foreign antigen stimulation in the presence of specific immunomodulatory properties. Other subtypes include interleukin-10 (IL-10)-secreting Tr1, transforming growth factor beta (TGF-β)-secreting T helper 3 (TH3) cells, CD4^+^Vα14^+^ NK Tregs, ɣδ T cells, and CD3^+^CD4^-^CD8^-^ (DN) Tregs [10,11].

Recently CD8^+^ suppressor T cells emerged as a novel entity of Tregs [8,10]. Several CD8^+^ suppressive T cell populations were reported; those expressing FoxP3 and CD25 were conventionally considered CD8^+^ Tregs [2,10, 24–26].

To date the phenotypic characterization of CD8^+^ Tregs and their role in the pathogenesis of autoimmune diseases in respect to the identified CD4^+^CD25^+^Foxp3^+^ counterpart remains to be fully unraveled [14]. According to Churlaud et al (2015) [10] CD8^+^ Tregs represents approximately 0.4% of T cells within the PBMC pool in healthy human subjects. CD8^+^ Tregs express low levels of CD127 consistently with CD4^+^ Tregs. Furthermore, CD8^+^ Tregs express lower levels of activation markers as CTLA-4, ICOS and ki-67 than in other CD8^+^ T cell subsets. Regarding CD25, the question of Treg sensitivity to IL-2 is of upmost importance to fully unravel the utility and applicability of their robust suppressive activity in clinical settings [10,22,27–28]. Tregs upon low-dose IL-2 treatment expressed enhanced levels of activation markers, including CD25, GITR, CTLA4 and basal phosphorylation of STAT5 (pSTAT5). Tregs retained a 20-fold higher sensitivity to IL-2 than T effector and NK cells [22]. Interestingly CD8^+^ Tregs were more prone to expand to low IL-2 dose treatment than CD4^+^ Tregs [10]. In addition subsets were identified as CD8^+^CD28^-^ Tregs [29–32], CD8^+^CD75s^+^ [33], plasmacytoid dendritic cell (DC2)-induced CD8 [34], CD8^+^CD45RC^high^ Tc1 Treg [35] and TCR peptide specific CD8αα Treg [36]. CD8^+^CD45RC^low^ T cells non-cytotoxic Tregs were reported, producing type 2 cytokines, expressing FoxP3 and CTLA-4 and able to counteract alloreactive immune responses. Their suppressive effect is mainly exerted through inhibition on CD4 T helper 1 (Th1) cells expansion and on their IFN-ɣ production [11].

As highlighted in the Introduction, the functionality of CD4^+^CD25^high^, either Foxp3 or CD127^low^, and effects on Teffs function has been intensively investigated by several authors in T1D patients (rev in [2, 37]). We observed defects of the CD4^+^ population activation in long-term patients due to a lower expression of PD-1 on their surface.

In this manuscript, no significant differences were depicted in the relative percentages of CD8^+^ Tregs and CD8^+^ Teffs among LT, ND T1D patients *versus* healthy normal controls in basal conditions as opposite to the results we published on CD4^+^ Tregs and CD4^+^ Treg/Teff ratio within PBMC that were found higher in the LT disease patients than in normal controls in basal conditions.

In basal conditions, no differences were observed in total percentages of CD8^+^, CD4^+^ T cells across the groups of ND, LT diabetics *versus* normal controls. We further confirmed the finding of significantly lower percentages of CD8^+^CD25^+^ T cells in the peripheral blood of LT diabetics than in healthy controls suggestive of a lower level of activation [2].

We analyzed the expression of PD-1, indicative of the performance of the PD-1/PDL-1 ligand regulatory pathway, on immunotypes and in particular, CD8^+^ Tregs, as previously unraveled for the CD4^+^CD25^+^ subset. PD-1 signaling is indeed required for the maintenance of functional CD4^+^CD25^+^FoxP3^+^ Tregs to control autoimmunity [38]. In CD4^+^ Tregs PD-1 expression was found inversely correlated with FoxP3 expression; further, low levels of PD-1 are necessary for the regulatory capacity of CD4^+^ Tregs, their TGF-β significant secretion and apoptosis resistance. Nevertheless very low levels of PD-1 expression can affect regulatory function, which relies on low levels of PD-1 expression, but not its absence [38]. Further experimental evidences in NOD mice demonstrate that PD-1 deficiency or administration of a monoclonal antibody to PD-1 promote and exacerbate diabetes development [39–41] with more pronounced CD4^+^ and CD8^+^ infiltration within insulitis. The initial study of Tsutsumi *et al*. [42] observed a decreased PD-1 expression in CD4^+^ T lymphocytes of a small and heterogeneous group of T1D patients as compared to healthy controls, suggesting that PD-1 plays a role in the development and maintenance of the disease. In a recent report by Fujisawa *et al*. [43] a lower PD-1 expression in CD4^+^ T cells contributed to the development of T1D through T cell activation. Significantly, a lower PD-1 expression was found in patients with the 7785 C/C genotype. There was no significant correlation between disease duration and frequency of PD-1 expression.

Regarding CD8^+^ Tregs, in basal conditions, we did not detect differences in the percentages of subsets expressing PD1, either total PD1^+^, PD1^high^ and PD-1^low^ expressing cells across ND, LD T1D patients and normal controls. Conversely lower percentages of Teff CD8^+^PD-1^+^, CD8+PD-1^high^ and CD8+PD-1^low^ cells were detected in LT patients than in normal controls indicative of their reduced efficiency and regulability.

For functional assessment, we adopted a polyclonal stimulation through PMA and ionomycin (S6 Fig). We interestingly observed reduced total percentages of CD8^+^ Tregs in LT diabetics together with increased percentages of CD8^+^ Teffs in the same group of patients respect to controls, confirming the occurred unbalance. Accordingly, Treg/Teff ratio was diminished in long-term diabetics *versus* controls. Furthermore, this ratio was also reduced in respect to newly diagnosed patients. Remarkably, the detection of similar percentages of total CD8^+^, CD4^+^ and CD8^+^CD25^+^subsets following stimulation comes further in support of their impaired efficacy whilst similar viability of CD8^+^ Treg and Teff populations. Considering the characteristics of polyclonal stimulation, which assesses the overall performance of CD8^+^ T cells, the defect is unmasked in particular for Teffs involved in general adaptive immune responses i.e. against non-self antigens and pathogens. In this regard, increased susceptibility to infections and vaccination failure has been observed in diabetes patients [44–48] not exclusively related to hyperglycemia. Indeed, abnormal immune responses and reduced CD4+ T cell proliferation have been postulated to occur in T1D patients [49–50].

Interestingly, frequency of CD8^+^ proliferating Tregs was reduced in ND diabetics *versus* healthy controls after 3 days of PMA-ionomycin stimulation. This reduction of CD8^+^ proliferating Tregs frequency was also observed among ND diabetics *versus* controls after 5 days of stimulation and, in addition, this phenomenon was observed for LT diabetics *versus* controls. At this time of stimulation, total frequency of proliferating CD8^+^ T cells was reduced in ND diabetics *versus* controls. Even CD8^+^ Teffs frequency was diminished in LT and ND diabetics *versus* healthy controls. Concurrently, of note, frequency of CD3^+^ and CD4^+^ proliferating T cells were similar among the 3 groups indicating that the reduced frequency results in the underlined subsets are not due to intrinsic increased level of apoptosis (S3 Fig).

Remarkably, in the functional evaluation, frequency of PD-1^+^CD8^+^ T cells and PD-1^high^ CD8^+^ T cells was reduced in LT diabetics *versus* healthy controls. Again total PD-1^+^ and PD-1^high^ CD8^+^ Tregs were significantly reduced in LT diabetics *versus* healthy controls. Conversely no significant difference was observed in the frequency of total PD-1^+^, PD-1^high^ and PD-1^low^ CD8^+^ Teff subsets.

As conclusive remark, our pilot study identifies a novel CD8^+^ Treg cell population, which is defective in diabetics due to a lower expression of PD-1 on its surface. As also applied to other subsets of Tregs the characterization of the CD8^+^ Treg population in the pancreas and peripheral lymphnodes of T1D patient should be also elucidated, although we can foresee the limitation of the lack of human biological specimens. Furthermore, a future research avenue could be to test their ‘antigen-specificity’ since these have been reported as more potent in suppressing autoimmunity than polyclonal Tregs [37].

Definitively our results encourage further studies on the role of PD-1/PD-L1 pathway in controlling the autoimmune process in T1D patients for tailored immunotherapeutic approaches to be developed in years to come.

## Supporting information

S1 Fig Representative gating strategy. Representative gating strategy for the flow cytometry analysis of lymphocytes for CD8^+^ T cell subsets. Data were collected with flow-cytometer Fortessa X-20analyzer (Becton and Dickinson (BD), Sunnyvale, CA, USA) and analyzed by FACSDiva software (BD Biosciences: San Jose, CA, USA). Lymphocytes were identified through their scatter properties (FSC-A×SSC-A plot). Due to the limited number of CD8^+^ Tregs, at least 20,000 CD8^+^ events were acquired. In this example, nitrogen frozen PBMC were thawed, stained as described in the method section for antibodies to CD3, CD8, CD25, PD-1 and Foxp3, and subsequently analyzed. The plots show depiction of CD8^+^CD25^+^ cells, CD8^+^ Tregs and CD8^+^ Teffs and the analysis of PD-1^+^, PD-1^high^ and PD-1^low^, between these last two subsets in RPMI (**a**) and after PMA/ionomycin stimulation (**b**).
(TIF)

S2 Fig Frequency of cell subsets under investigation relative to HD, ND and LT T1D patients upon three days unstimulated culture condition (RPMI). Flow-cytometry analysis of healthy donor and patients PBMC following three days of unstimulated RPMI culture. Graphs show the percentage of CD4^+^ (**a**), CD8^+^ (**b**), CD8^+^CD25^+^Foxp3^+^ Treg (**c**), CD8^+^CD25^-^Foxp3^-^ Teff (**d**) cells. For the investigation present in figure, 13 HD, 9 ND and 9 LT were studied.
(TIF)

S3 Fig Proliferative responses of the subsets under study in HD, ND and LT T1D patients after three days of PMA/ionomycin stimulation. CMFDA-labeled PBMC from HD and T1D patients were stimulated with PMA/ionomycin for three and five days and subsequently stained for flow-cytometry analysis. Graphs show the frequency of CD3^+^ (**a**), CD4^+^ (**b**) proliferating cells after 3 and 5 (**c-d**) days of stimulation. Proliferation was evaluated as percentage of CMFDA-low cells relative to the subset analyzed after stimulation over the percentage of CMFDA-low cells of the same subset in RPMI unstimulated culture. For the investigation present in figure, 15 HD, 9 ND and 9 LT were studied.
(TIF)

S4 Fig Correlation of percentages of CD8^+^ Treg cells with levels of HbA1c under basal conditions. (**a**) Analysis performed in ND T1D and (**b**) LT T1D patients. For the investigation present in figure, 18 ND and 13 LT samples were studied.
(TIF)

S5 Fig Correlation of percentages CD8^+^ PD-1^+^ Treg cells and percentages CD8^+^ PD-1^+^ Teff cells with levels of HbA1c under basal conditions. (**a**) Analysis performed for percentages of CD8^+^ Treg PD-1^+^ cells in ND T1D and (**b**) LT T1D patients; (**c**) Analysis performed for percentages of CD8^+^ Teff PD-1^+^ cells in ND T1D and (**d**) LT T1D patients. For the investigation present in figure, 18 ND and 13 LT samples were studied.
(TIF)

S6 Fig Viability of cell cultures after PMA/ ionomycin stimulation. (**a**) Histogram shows the percentage of viable lymphocytes after 3 days of PMA/ionomycin stimulation (Kruskal–Wallis one-way analysis of variance p <0.05). (**b**) Histogram shows the % of viable lymphocytes after 5 days of PMA/ionomycin stimulation (Kruskal–Wallis one-way analysis of variance p <0.05). For the investigation present in figure, 14 HD, 9 ND and 9 LT samples were studied.
(TIF)
