# Supplementary figures and images for: Identification and functional characterization of CD8+ T regulatory cells in type 1 diabetes patients
Source: PLoS One. 2019 Jan 16;14(1):e0210839. doi: 10.1371/journal.pone.0210839 (PMC6334945; doi:10.1371/journal.pone.0210839)

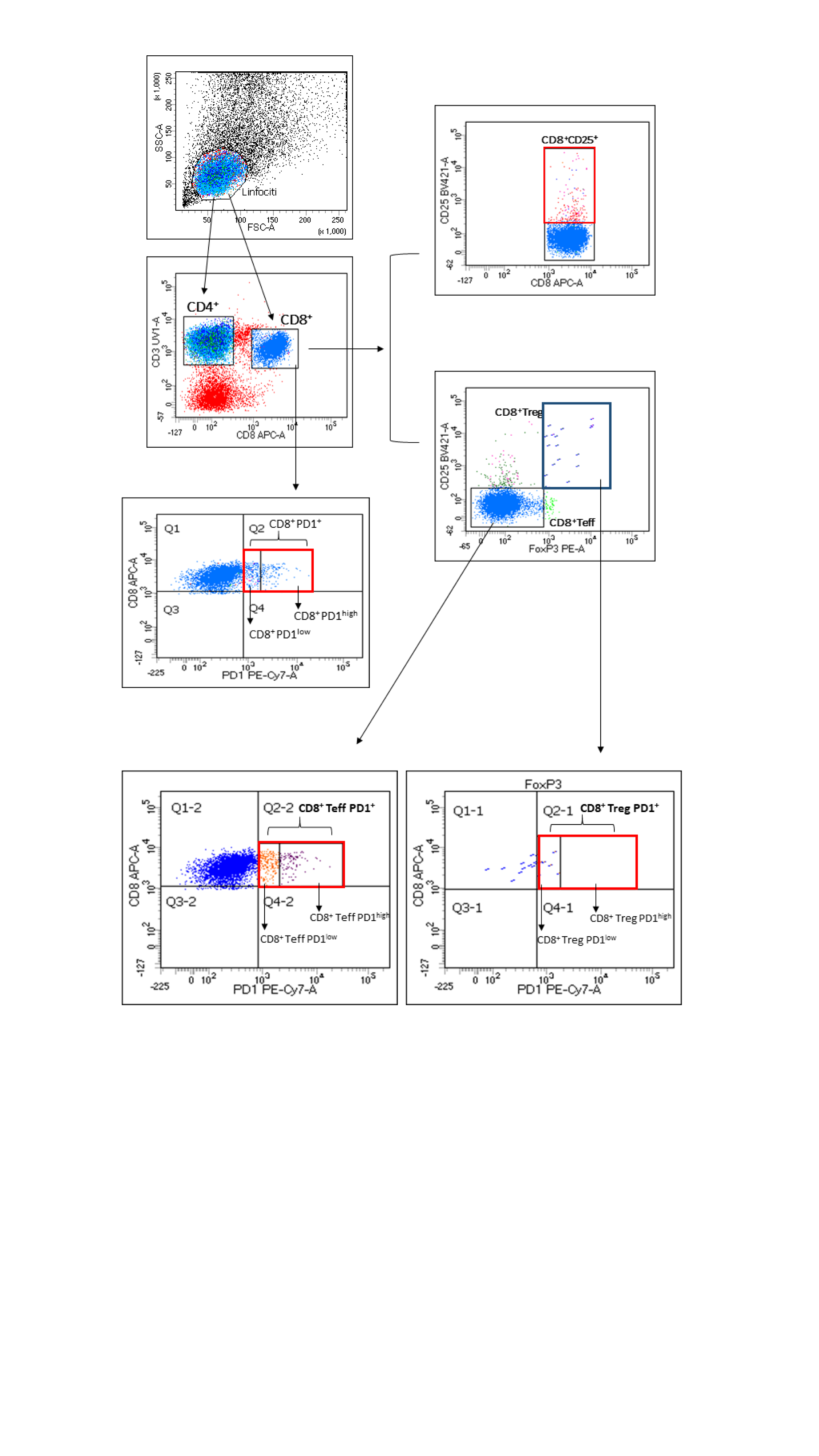

Supplement: S1 Fig — Representative gating strategy for the flow cytometry analysis of lymphocytes for CD8+ T cell subsets. Data were collected with flow-cytometer Fortessa X-20analyzer (Becton and Dickinson (BD), Sunnyvale, CA, USA) and analyzed by FACSDiva software (BD Biosciences: San Jose, CA, USA). Lymphocytes were identified through their scatter properties (FSC-A×SSC-A plot). Due to the limited number of CD8+ Tregs, at least 20,000 CD8+ events were acquired. In this example, nitrogen frozen PBMC were thawed, stained as described in the method section for antibodies to CD3, CD8, CD25, PD-1 and Foxp3, and subsequently analyzed. The plots show depiction of CD8+CD25+ cells, CD8+ Tregs and CD8+ Teffs and the analysis of PD-1+, PD-1high and PD-1low, between these last two subsets in RPMI (a) and after PMA/ionomycin stimulation (b). (TIF) [file pone.0210839.s001.tif]

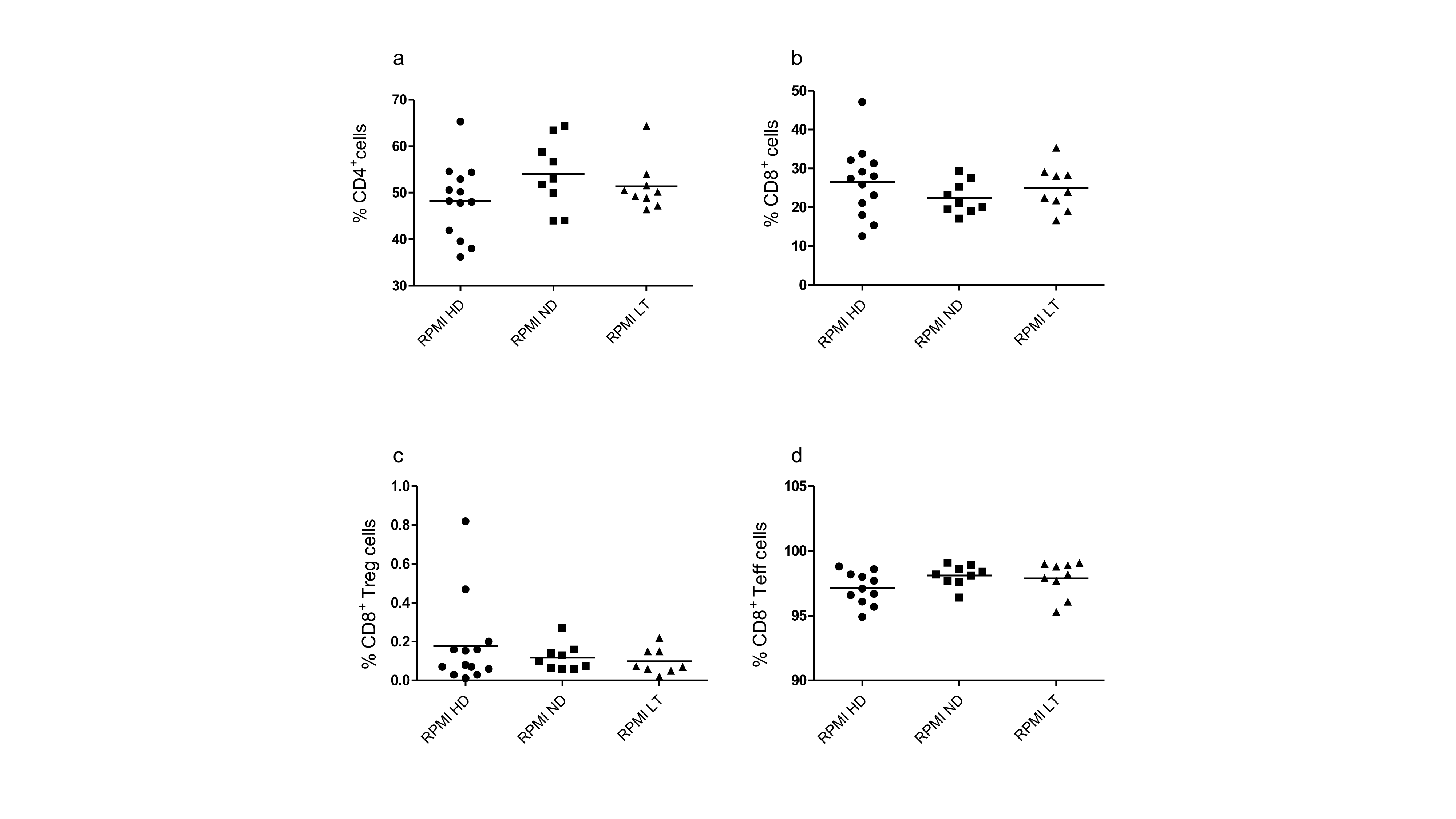

Supplement: S2 Fig — Flow-cytometry analysis of healthy donor and patients PBMC following three days of unstimulated RPMI culture. Graphs show the percentage of CD4+ (a), CD8+ (b), CD8+CD25+Foxp3+ Treg (c), CD8+CD25-Foxp3- Teff (d) cells. For the investigation present in figure, 13 HD, 9 ND and 9 LT were studied. (TIF) [file pone.0210839.s002.tif]

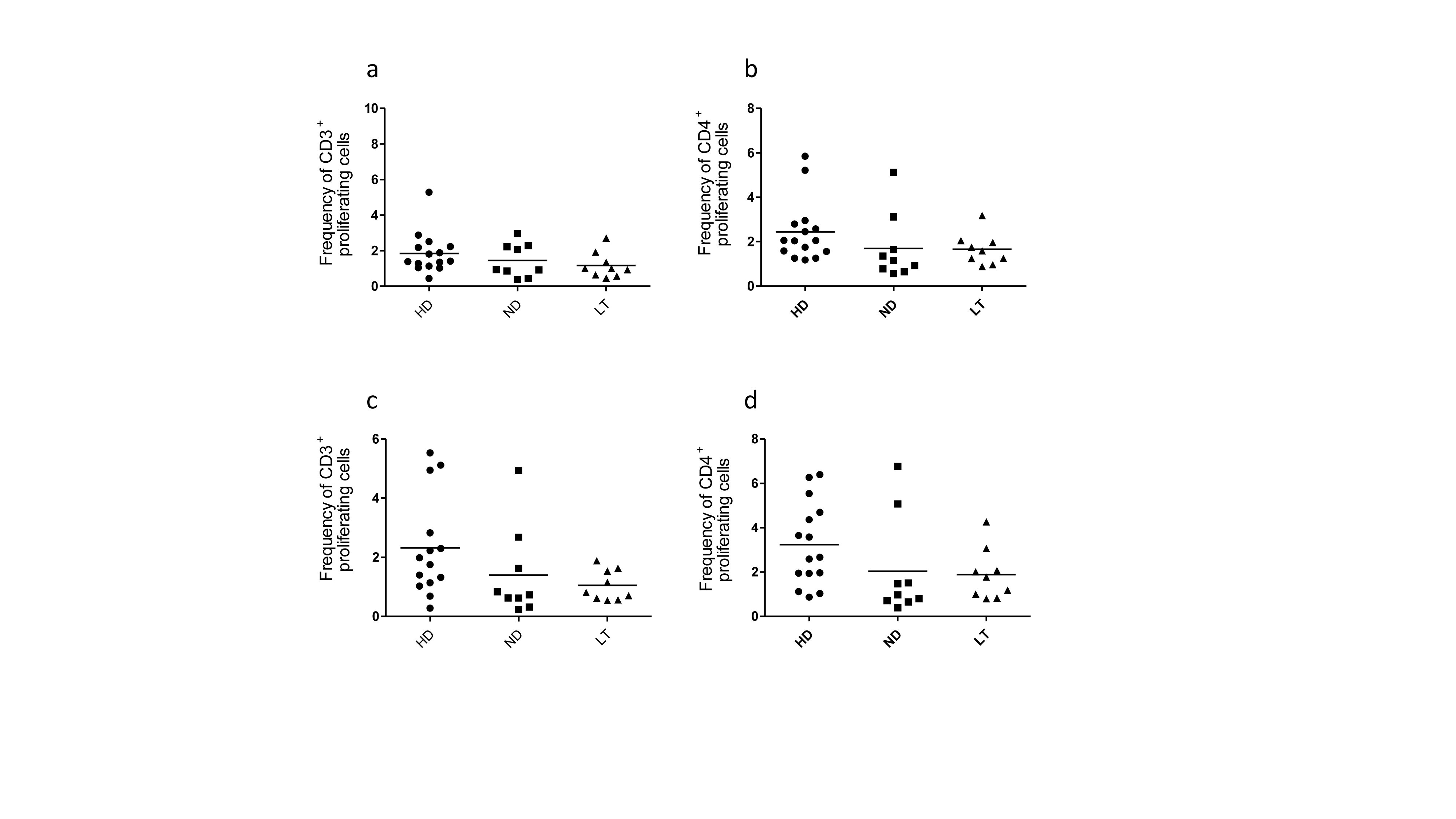

Supplement: S3 Fig — CMFDA-labeled PBMC from HD and T1D patients were stimulated with PMA/ionomycin for three and five days and subsequently stained for flow-cytometry analysis. Graphs show the frequency of CD3+ (a), CD4+ (b) proliferating cells after 3 and 5 (c-d) days of stimulation. Proliferation was evaluated as percentage of CMFDA-low cells relative to the subset analyzed after stimulation over the percentage of CMFDA-low cells of the same subset in RPMI unstimulated culture. For the investigation present in figure, 15 HD, 9 ND and 9 LT were studied. (TIF) [file pone.0210839.s003.tif]

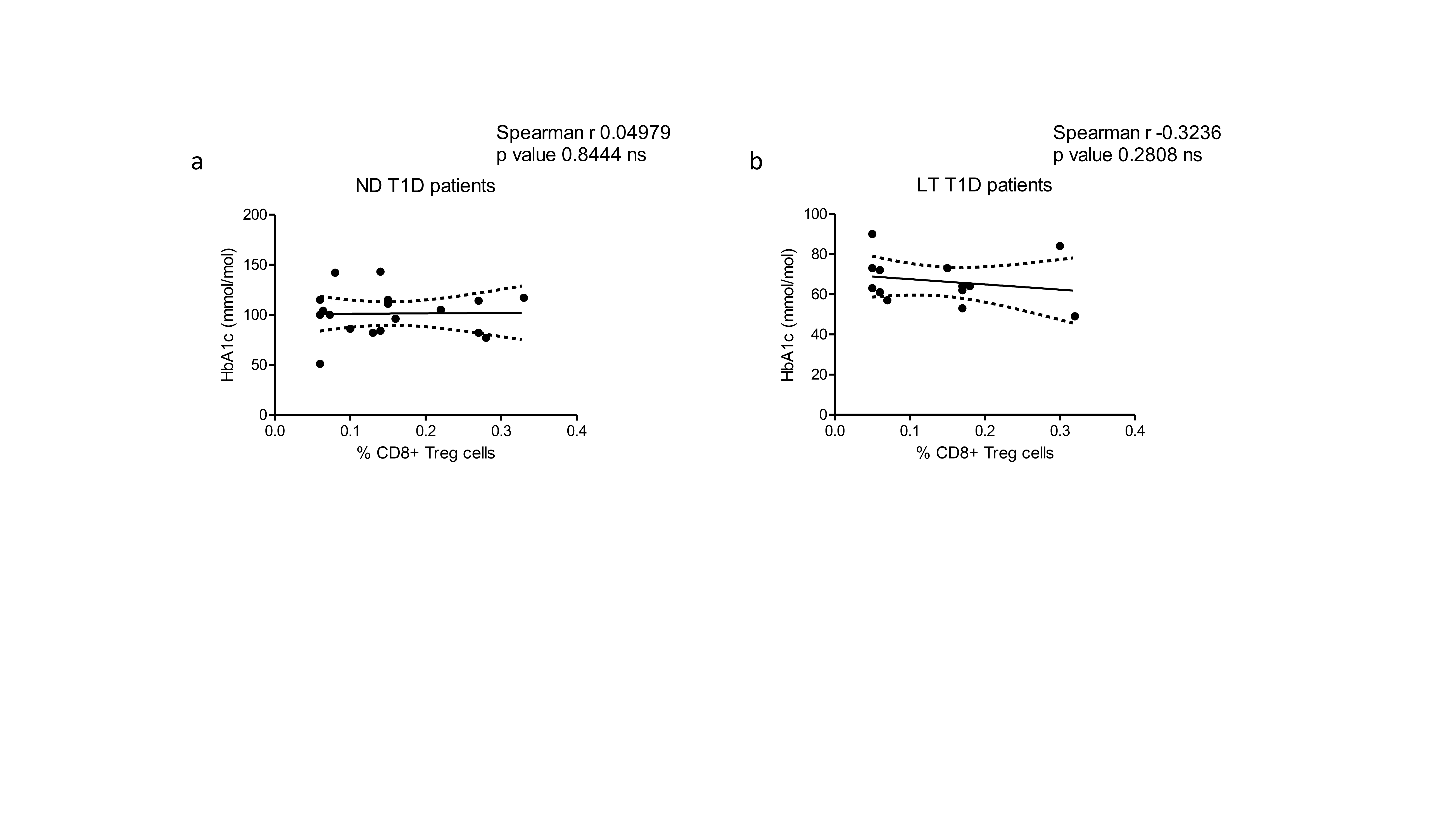

Supplement: S4 Fig — (a) Analysis performed in ND T1D and (b) LT T1D patients. For the investigation present in figure, 18 ND and 13 LT samples were studied. (TIF) [file pone.0210839.s004.tif]

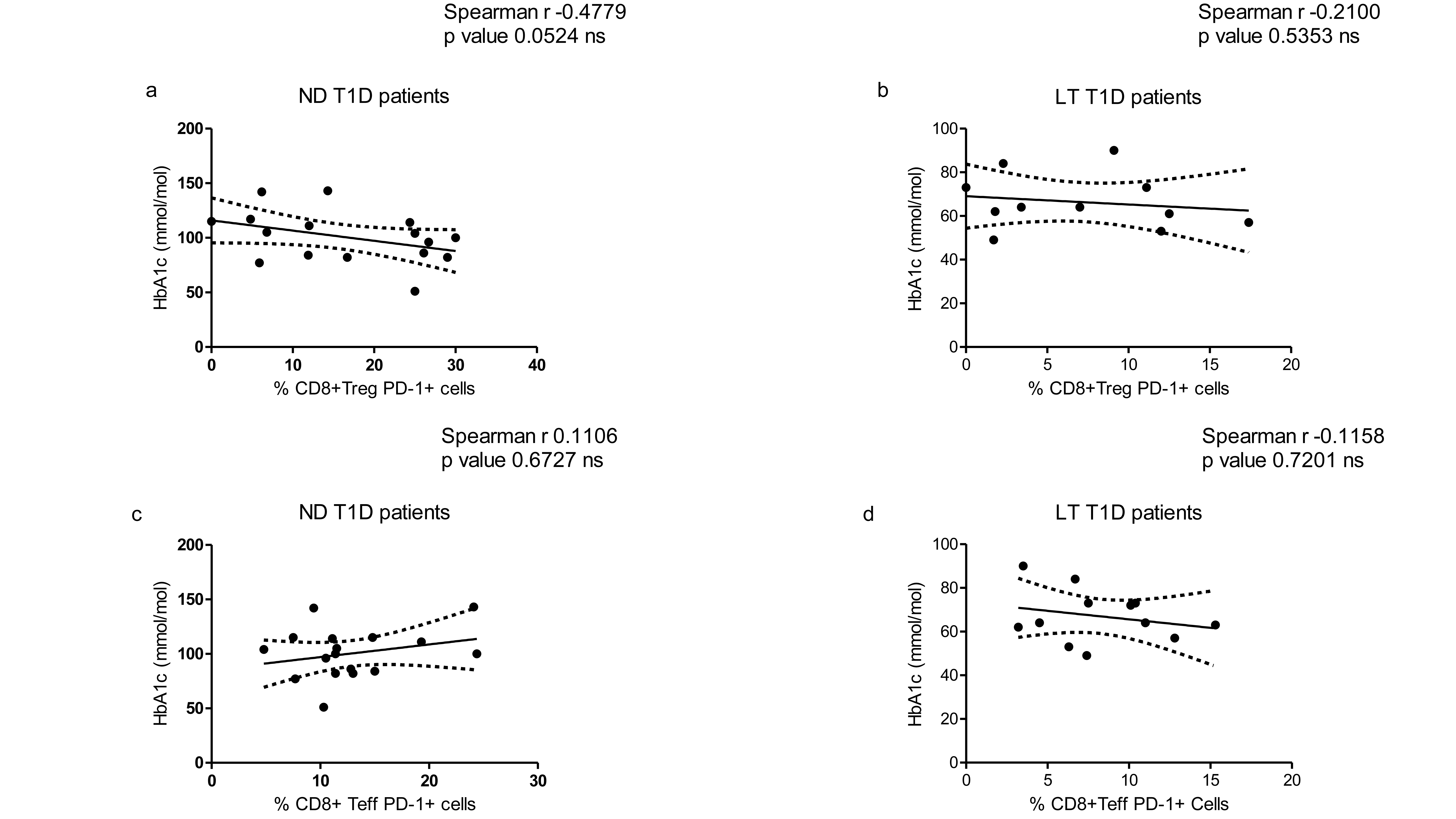

Supplement: S5 Fig — (a) Analysis performed for percentages of CD8+ Treg PD-1+ cells in ND T1D and (b) LT T1D patients; (c) Analysis performed for percentages of CD8+ Teff PD-1+ cells in ND T1D and (d) LT T1D patients. For the investigation present in figure, 18 ND and 13 LT samples were studied. (TIF) [file pone.0210839.s005.tif]

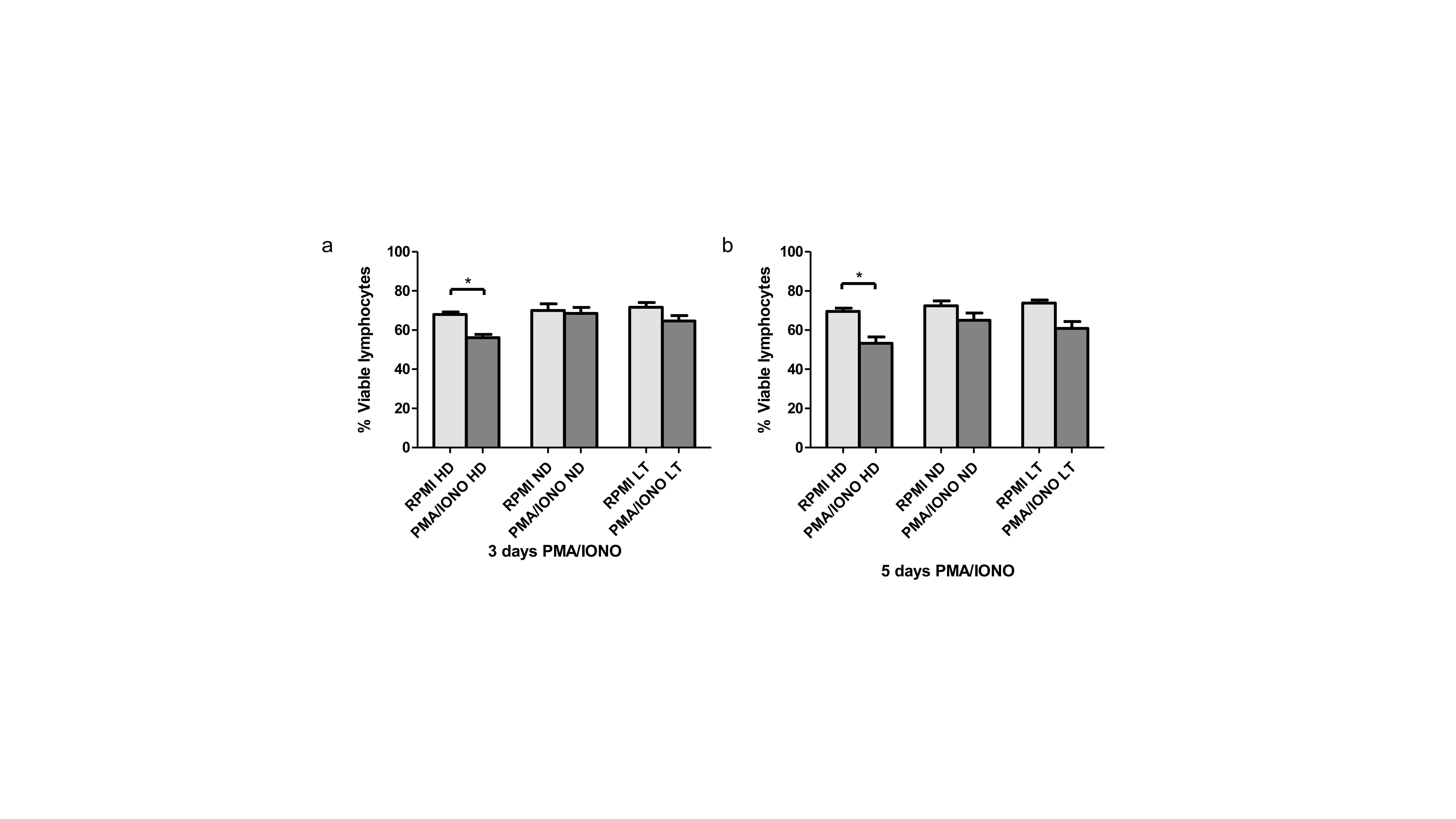

Supplement: S6 Fig — (a) Histogram shows the percentage of viable lymphocytes after 3 days of PMA/ionomycin stimulation (Kruskal–Wallis one-way analysis of variance p <0.05). (b) Histogram shows the % of viable lymphocytes after 5 days of PMA/ionomycin stimulation (Kruskal–Wallis one-way analysis of variance p <0.05). For the investigation present in figure, 14 HD, 9 ND and 9 LT samples were studied. (TIF) [file pone.0210839.s006.tif]
